# Analysis of molecular pathologic and clinical features of 36 patients with pulmonary sarcomatoid carcinoma

**DOI:** 10.1186/s12890-022-02248-9

**Published:** 2022-11-29

**Authors:** Yingying Yu, Xiumei Duan, Shuai Wang, Hua He, Shijie Lan, Zhen Guo, Di Wu

**Affiliations:** 1 Cancer Centre, First Hospital of Jilin University, Changchun, 130012 Jilin China; 2 Department of Comprehensive Oncology, People’s Hospital of Jilin Province, Changchun, 130012 Jilin China; 3 Pathology Department, First Hospital of Jilin University, Changchun, 130012 Jilin China; 4 Pathology Department, Jilin Province Tumor Hospital, Changchun, 130012 Jilin China

**Keywords:** Next generation sequencing, Pulmonary sarcomatoid carcinoma, Genetic mutation, Molecular characterization, Clinical features

## Abstract

**Background:**

Pulmonary sarcomatoid carcinoma (PSC) is a heterogeneous disease with poor prognosis. It is essential to understand the molecular basis of its progression in order to devise novel therapeutic strategies. The aim of this study was to identify the pathological mutations in PSC through next generation sequencing technology (NGS), and provide reference for the diagnosis and molecular targeted therapy.

**Materials and methods:**

Thirty-sex patients with pathologically confirmed PSC who underwent surgical tumor resection at The First Hospital of Jilin University and Jilin Cancer Hospital from June 2011 to June 2017 were enrolled. Thirteen patients were successfully followed up and detailed clinical data were obtained. NGS was performed for the exons of entire oncogenes. Kaplan–Meier method was used for the univariate analysis, and the Cox proportional risk regression model was used for multivariate analysis.

**Results:**

A total of 19 highly frequent mutations were identified, of which the KRAS, BRCA1 and ALK mutations were significantly correlated with the overall survival (OS). Multivariate analysis showed that KRAS mutation was an independent factor affecting the OS of PSC patients.

**Conclusion:**

The KRAS mutation is an independent prognostic factor for PSC, and patients harboring the KRAS mutation had significantly shorter OS compared to patients with wild type KRAS. The characteristic mutation landscape of PSC may guide clinical targeted therapy.

Pulmonary sarcomatoid carcinoma(PSC) is a rare type of non-small cell lung cancer(NSCLC) with sarcomatoid cell or sarcomatoid (spindle/giant cell) differentiation, and accounts for about 0.1–0.4% of all NSCLC cases [1]. In the 2015 edition, WHO classified PSC into 5 subtypes, including pleomorphic carcinoma, spindle cell carcinoma, giant cell carcinoma, carcinosarcoma, and pulmonary blastoma [2]. Pleomorphic carcinoma is the most common subtype, and is defined as a class of poorly differentiated NSCLC, i.e., squamous cell carcinoma, adenocarcinoma or large cell carcinoma containing at least 10% spindle cells and/or giant cells [3]. Spindle cell carcinoma consists only of spindle cells that are tightly nested and arranged in irregular bundles, and resemble those seen in pleomorphic carcinoma [4]. Giant cell carcinoma is composed exclusively of giant cells, including polymorphic mononuclear and/or multinucleated neoplastic giant cells. Unlike the giant cells observed in pleomorphic carcinoma, those in giant cell carcinoma are mostly dispersed [4]. Carcinosarcoma is a mixed malignancy consisting of NSCLC cells (mainly squamous or adenocarcinoma cells) and sarcomatous cells (rhabdomyosarcoma, chondrosarcoma and osteosarcoma cells), and has bidirectional differentiation characteristics. Carcinosarcoma and pleomorphic carcinoma are primarily distinguished on the basis of these heterologous components [4]. Squamous cell carcinoma is the most common type of epithelial tumor, followed by adenocarcinoma and adeno-squamous carcinoma. The most common mesenchymal tumor is rhabdomyosarcoma, followed by osteosarcoma and chondrosarcoma [5]. Pulmonary blastoma is a rare malignancy comprising of primitive epithelial and mesenchymal cells similar to well-differentiated fetal adenocarcinoma.

PSC patients do not exhibit any obvious clinical symptoms at the early stage, and most of them are diagnosed at the advanced stage that precludes the possibility of surgery. In addition, PSC is recalcitrant to the conventional chemotherapeutic drugs for lung cancer, which seriously affects the quality of life and prognosis of patients [1]. In this study, we performed high-throughput sequencing on the tumor specimens collected from 36 PSC patients to identify mutations in the oncogene exons. The influence of the clinical characteristics and gene mutations on the overall survival (OS) of the patients were analyzed to identify possible therapeutic targets.

## Materials and methods

### Pathological-diagnosis

The patients were diagnosed on the basis of the 2015WHO edition [2].

### NGS platform

The whole sequencing reaction of NGS is carried out on the chip, which can rapidly generate millions of DNA and RNA fragments in a short time, and sequence millions of sites at the same time [6]. With the continuous development of this technology, the advantages in accuracy, sensitivity, price and other aspects continue to show. At present, NGS is widely used in the field of human life science research, which is of great significance in further understanding the relationship between gene mutations and human health and diseases.

### Patients

Using the case retrieval system databases of the First Hospital of Jilin University and Jilin Cancer Hospital, 17 and 19 patients who respectively underwent surgical resection from June 2011 to June 2017 were selected. Tumor staging was established according to the seventh edition of the AJCC guidelines, and the clinical data of the patients was retrieved (Table 1). Formalin-fixed paraffin-embedded tumor tissue blocks were obtained, and 10 slices measuring 10-μm in thickness were prepared for each patient. Kalpan-Meier univariate analysis and COX multivariate analysis were performed on 13 patients with complete follow-up information, and 23 patients with missing follow-up information were excluded.

**Table 1:** Clinical features of 36 PSC patients

Variable	Total (%)
*Sex*	
Male	25 (69.4)
Female	11 (30.6)
*Age*	
> 60	21 (58.3)
≤ 60	15 (41.7)
*Smoking status*	
Smkers	26 (72.2)
Nonsmkers	10 (27.8)
*Tumor size*	
> 5 cm	16 (44.4)
≤ 5 cm	20 (55.6)
*Tumor site*	
Left	18 (50.0)
Right	18 (50.0)
*T stage*	
T1 and T2	28 (77.8)
T3 and T4	8 (22.2)
*N stage*	
N0 and N1	28 (77.8)
N2 and N3	8 (22.2)
*Clinical stage*	
Ia and IIa	21 (58.3)
IIb and IIIb	15 (41.7)
*Pathological type*	
Pleomorphic carcinoma	31 (86.1)
other	5 (13.9)
*Treatment*	
Surgery	24 (66.7)
Surgeryand chemotherapy	12 (33.3)

### Follow-up

Thirty-six patients were followed up by telephone interviews, and the last follow-up was conducted on January 30, 2018. Twenty-three were lost during the follow-up, and the prognostic information of 13 patients were subsequently analyzed. Survival duration was calculated from the time of diagnosis to that of the last follow-up or death.

### Statistical analysis

The data were analyzed by SPSS 22.0 statistical software. Univariate survival analysis was performed by the Kaplan–Meier method, and Log-rank test was used to compare the groups (test level a = 0.05). Cox proportional risk regression model was used for multivariate survival analysis. *P* < 0.05 was considered statistically significant.

## Results

### Identification of prognostic factors of PSC

Univariate analysis of 13 patients that were successfully followed-up showed that gender, age, smoking status, tumor diameter, tumor location, clinical stage, pathological classification and treatment were not significantly correlated to the OS (*P* > 0.05).

Multivariate Cox regression further showed that gender, age, smoking status, tumor diameter, tumor location, T stage, N stage, clinical stage, pathological classification and treatment were not independent factors affecting the OS of PSC patients (*P* > 0.05).

### Pathological characteristics

Immunostaining of the tumors specimens showed that 100% of the patients were positive for CK and Vimentin, while the positive rates for CK5/6, CK7, TTF-1 and P63 were 36.1%, 63.9%, 63.9% and 47.2% respectively (Table 2).

**Table 2:** Immunohistochemical characteristics

Immunophenotype	Total (%)
CK	36 (100)
CK5/6	13 (36.1)
CK7	23 (63.9)
Vimentin	36 (100)
TTF-1	23 (63.9)
P63	17 (47.2)

**Fig. 1:**
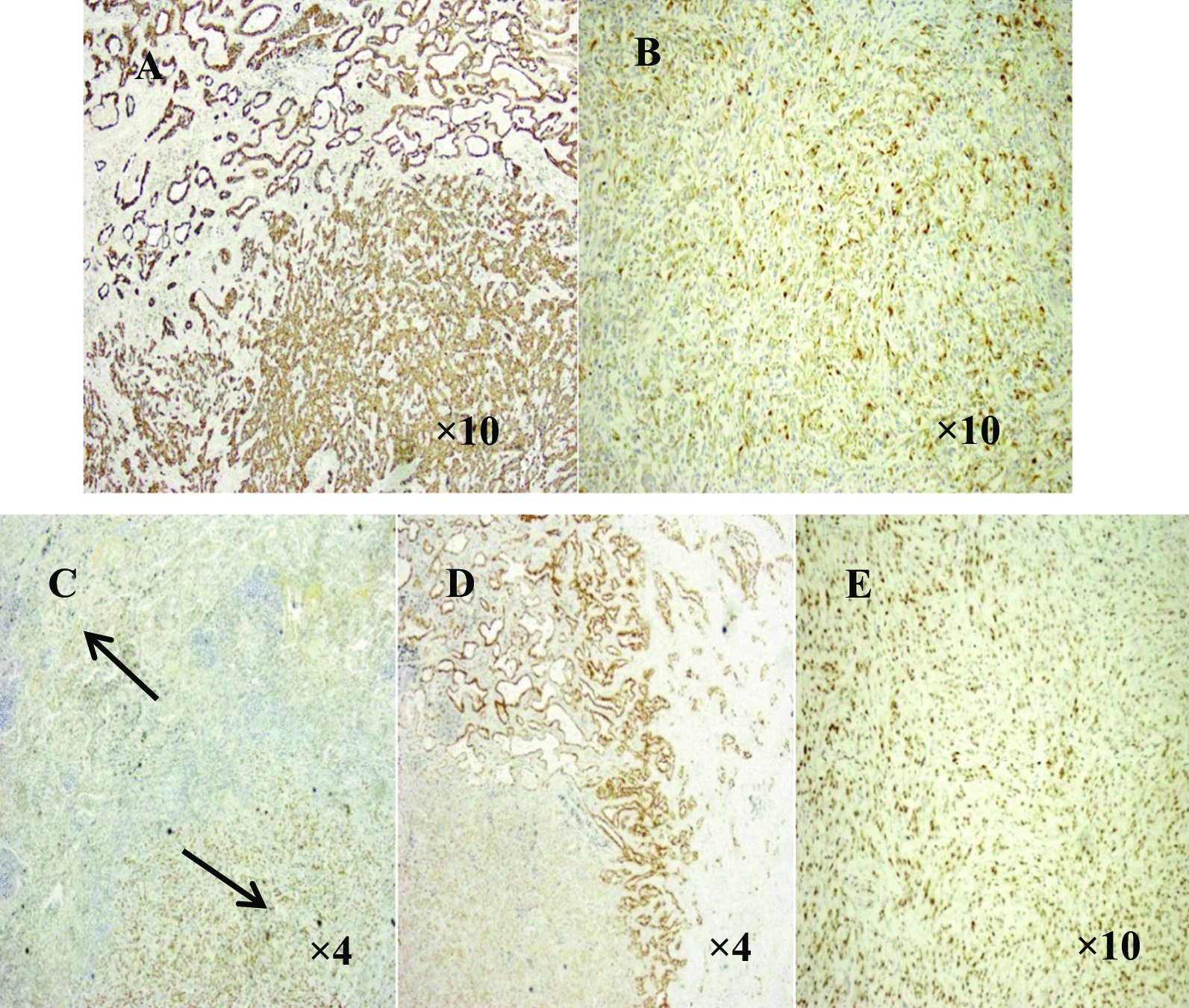
Immunohistochemical characteristics of pleomorphic carcinoma **A** CK7 positive (HE, × 10); **B** Vimentin spindle composition expression (HE, × 10) **C** P63 squamous carcinoma expression (the lower right), adenocarcinoma of the negative (the upper left) (HE, × 10); **D** TTF-1 adenocarcinoma components expression, squamous cell carcinoma components negative (HE, × 4); **E** TTF-1 spindle composition expression (HE, × 10)

**Fig. 2:**
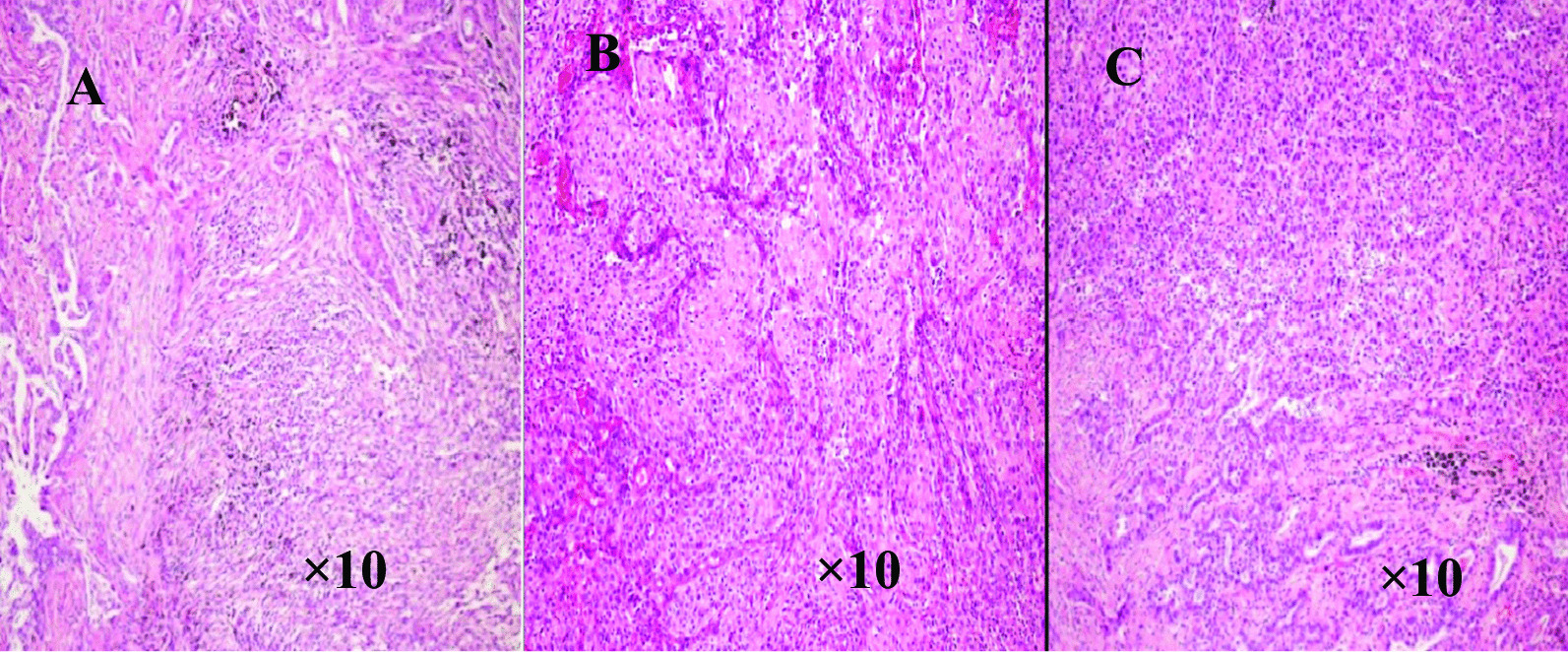
Histopathological features of pleomorphic carcinoma **A** adenocarcinoma and spindle cell components (HE, × 10); **B** squamous cell carcinoma components (HE, × 10); **C** squamous cell carcinoma and adenocarcinoma components (HE, × 10)

**Fig. 3:**
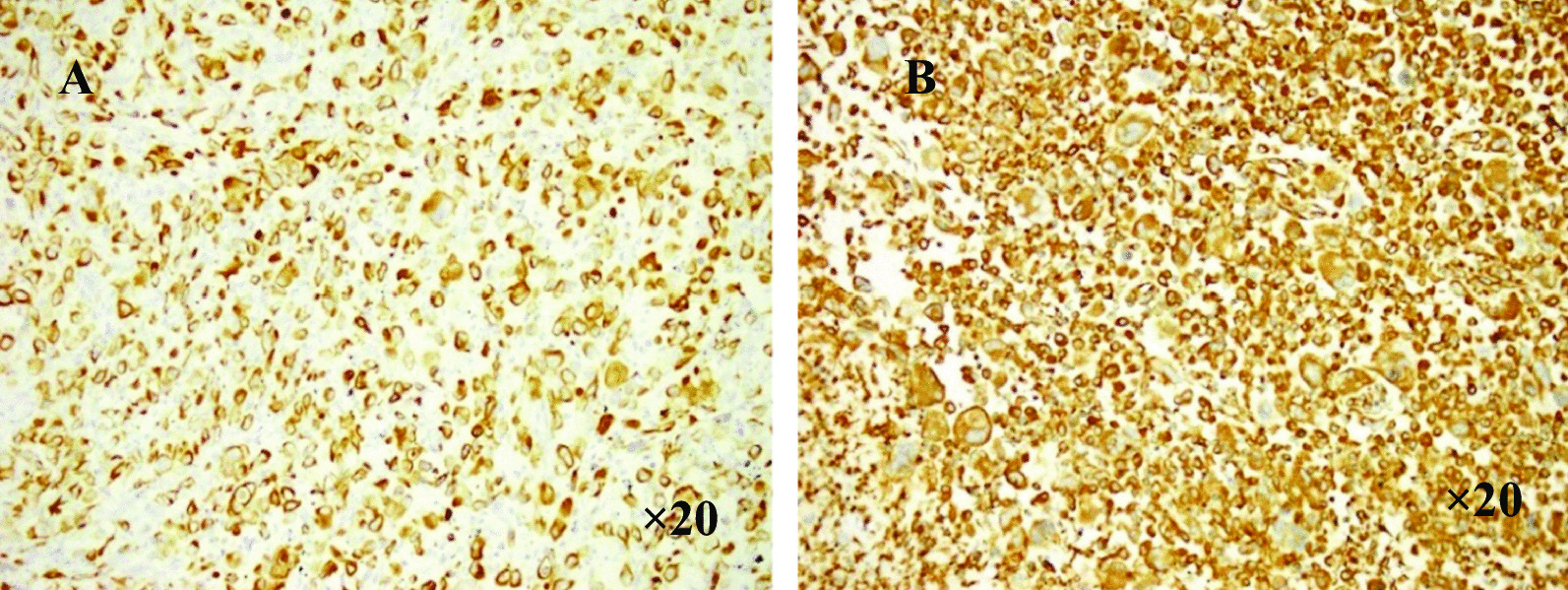
Immunohistochemical characteristics of giant cell carcinoma **A** CK7 positive (HE, × 20); **B** Vimentin positive (HE, × 20)

**Fig. 4:**
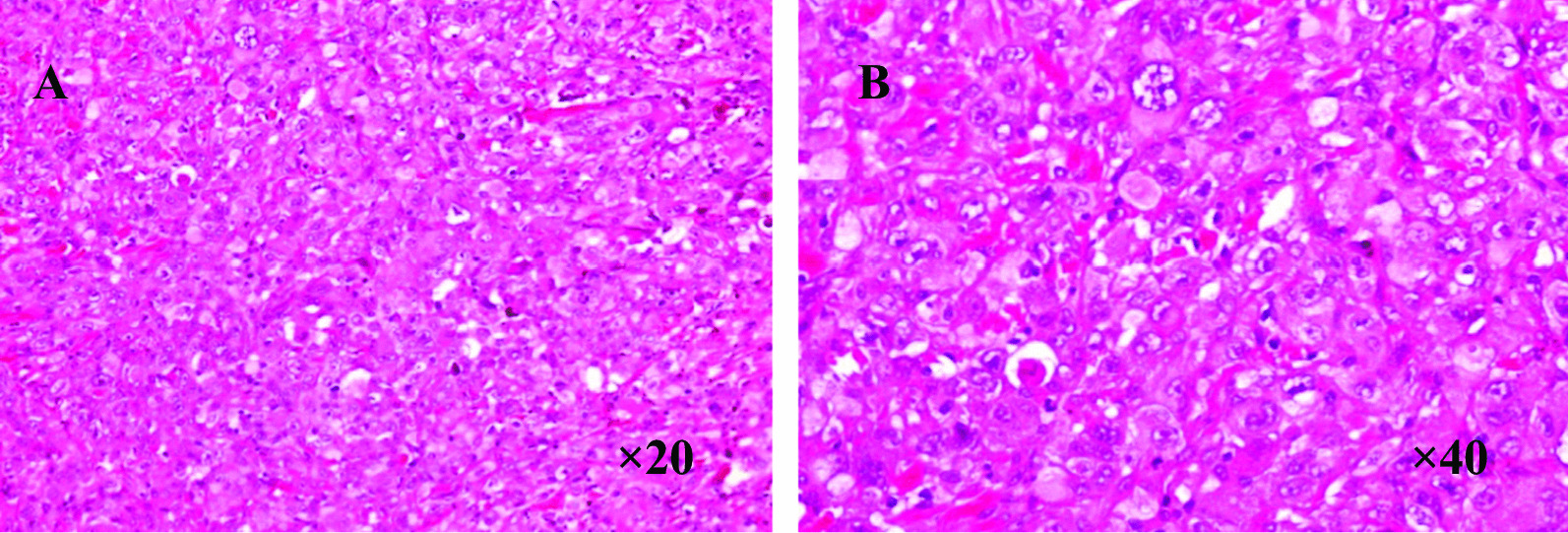
Histopathological features of giant cell carcinoma **A** Giant cell carcinoma consists of mononuclear neoplastic giant cells (HE, × 20) **B** Giant cell carcinoma (HE, × 40)

The immunophenotypes are the result of both differentiated and undifferentiated cells (Fig. 1). Histopathological examination indicated presence of squamous cell carcinoma, adenocarcinoma and at least 10% spindle cells (Fig. 2). The giant cell carcinoma specimens were positive for both CK7 and Vimentin (Fig. 3), and comprised entirely of polymorphic mononuclear and/or multinuclear neoplastic giant cells (Fig. 4).

### Frequency of gene mutations

NGS revealed 19 high-frequency mutations, including TP53 (69.4%), PKD1 (38.9%), THADA (33.3%), RB1 single copy deletion (30.6%),KRAS (30.6%), PIK3CA (25.0%), EGFR (19.4%), NF1 (16.7%), PTCH1 (16.7%), BRCA1 (16.7%), BRAF (16.7%), ARID1A (16.7%), mTOR (16.7%), MET (13.9%), CREBBP (11.1%), ARID2 (11.1%), ALK (11.1%), CDK12 (8.3%) and EMLA4-ALK fusion (5.6%).

### Association between gene mutation and OS

**Fig. 5:**
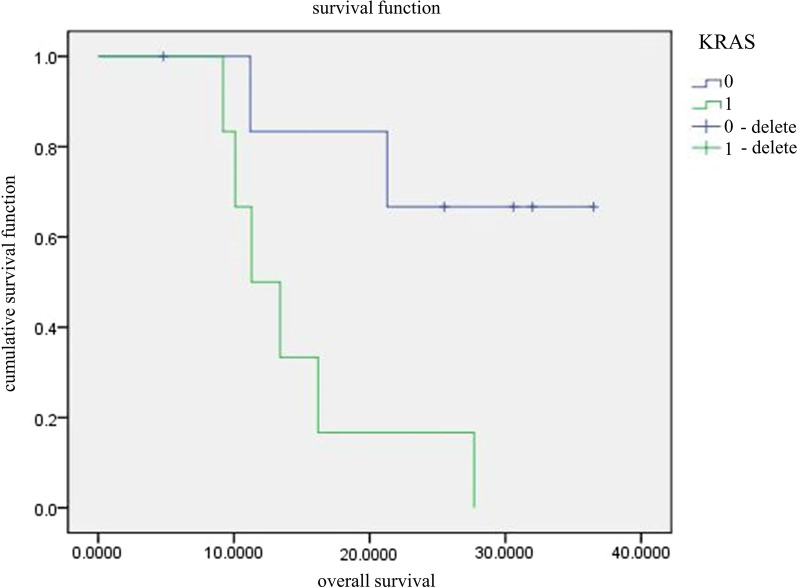
Comparison of survival curves between KRAS^mutant^ and KRAS wild-type

**Fig. 6:**
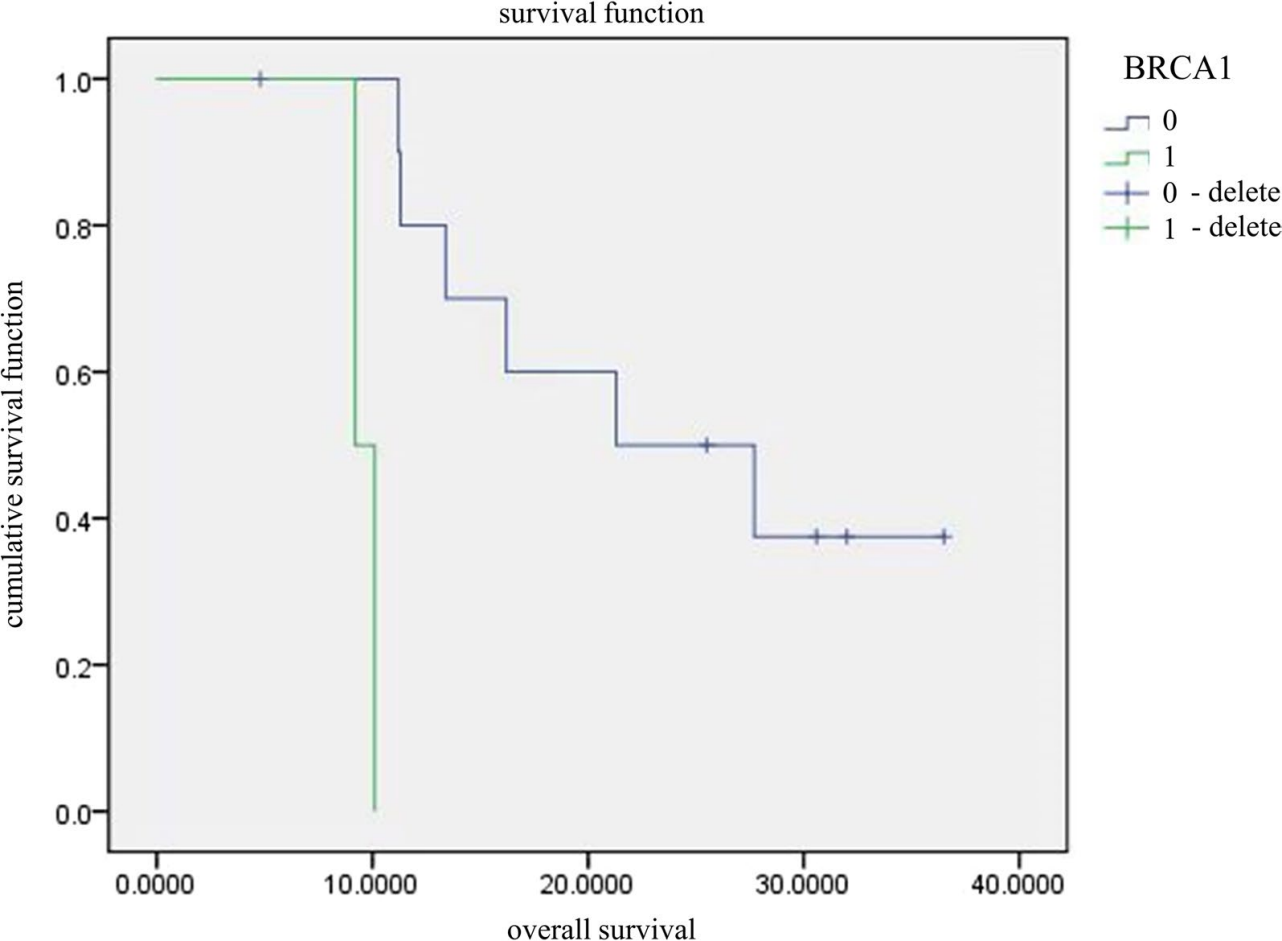
Comparison of survival curves between BRCA1^mutant^ and BRCA1 wild-type

Of the 13 patients that were followed up, 6 harbored KRAS mutations and 7 patients had the wild-type KRAS, and the KRAS^mutant^ group had significantly worse OS compared to the wild-type controls (*P* = 0.018; Table 3; Fig. 5). There were 2 patients with BRCA1 mutation and 11 patients with BRCA1 wild-type, and the mutation significantly affected patient prognosis (P < 0.0001; Table 3; Fig. 6). However, given the miniscule size of the BRCA1^mutant^ group, the above results need further clinical validation. Only one patient had an ALK mutation, which was associated significantly with the OS (P = 0.038; Table 3), although its clinical relevance needs clarification. No BRAF and MET mutations were detected among the 13 patients, while TP53, PKD1, THADA, RB1 single copy deletion, PIK3CA, EGFR and NF1, PTCH1 and ARID1A, MTOR, CREBBP, ARID2, CDK12 and EMLA4-ALK fusion had no significant effect on the OS (P > 0.05).

**Table 3:** Univariate survival analysis of gene mutation

Variable	*p* Values
TP53	0.913
PKD1	0.284
THADA	0.506
RB1 single copy deletion	0.532
KRAS	0.018^a^
PIK3CA	0.908
EGFR	0.456
NF1	0.261
PTCH1	0.115
BRCA1	0.000^a^
ARID1A	0.178
MTOR	0.590
CREBBP	0.649
ARID2	0.297
ALK	0.038^a^
CDK12	0.261
EMLA4-ALK fusion	0.261

^a^*p* < 0.05

Cox proportional risk regression model further showed that the KRAS mutation was an independent prognostic for PSC (Table 4).

**Table 4:** Multivariate survival analysis of gene mutation

Variable	B	SE	*Pvalues*	Exp(B)	95%CI
TP53	−0.119	1.084	0.913	0.888	0.106–7.438
PKD1	0.782	0.745	0.294	2.185	0.507–9.416
THADA	0.486	0.738	0.510	1.626	0.383–6.908
RB1 single copy deletion	−0.659	1.073	0.539	0.518	0.063–4.236
KRAS	1.779	0.839	0.034^a^	5.921	1.143–30.64
PIK3CA	−0.085	0.738	0.908	0.918	0.216–3.902
EGFR	−0.782	1.074	0.467	0.458	0.056–3.756
NF1	−3.259	4.561	0.475	0.038	0.00–293.04
PTCH1	1.285	0.871	0.140	3.616	0.655–19.96
BRCA1	9.870	35.918	0.783	19,334.66	5.165E−27–7.238E + 34
ARID1A	−1.351	1.077	0.210	0.259	0.031–2.136
MTOR	−0.443	0.828	0.593	0.642	0.127–3.255
CREBBP	0.495	1.099	0.652	1.641	0.190–14.140
ARID2	1.142	1.156	0.323	3.134	0.325–30.215
ALK	2.350	1.415	0.097	10.488	0.656–167.810
CDK12	−3.259	4.561	0.475	0.038	0.000–293.024
EMLA4-ALKfusion	0.446	1.073	0.677	1.562	0.191–12.798

^a^*p* < 0.05

## Discussion

PSC is a rare lung malignancy that accounts for about 2%-3% of all NSCLC cases [1, 7]. The obvious heterogeneity of PSC not only increases the difficulty of clinical diagnosis, but also decreases the efficacy of conventional therapies [8, 9]. Therefore, it is crucial to identify novel prognostic factors and therapeutic factors to improve patient prognosis. The identification of panoramic oncogene exons through NGS has accelerated the development of molecular targeted therapies for lung adenocarcinoma, malignant melanoma, breast cancer and other tumors based on specific gene mutations. This approach is particularly effective for screening mutations associated with rare malignancies, such as PSC.

In this study, the average age of the PSC patients was 60 years, which is consistent with previous reports indicating that PSC is more prevalent in middle-aged and elderly men with 60–66 years of age [1]. In addition, previous studies have shown that clinicopathological features such as tumor diameter > 5 cm, clinical stage > I and lymph node invasion portend worse survival in PSC patients, and early surgical treatment may improve prognosis [10]. However, we did not detect any significant association of smoking status, tumor diameter, clinical stage and surgical treatment with the survival of PSC patients.

Mutations in TP53, PKD1, THADA, RB1 (single copy deletion), KRAS, PIK3CA, EGFR, NF1, PTCH1, BRCA1, BRAF, ARID1A, mTOR, MET, CREBBP, ARID2, ALK, CDK12 and EMLA4-ALK (fusion) were frequent in the tumor specimens obtained from 36 PSC patients. Previous reports on the frequency of gene mutations in PSC were largely focused on the European population, and the mutations were mostly sporadic. Almost 50% of the NSCLC patients harbor mutations in the TP53 gene, which is closely related to the occurrence and development of tumors [11]. However, the frequency of TP53 mutations in our cohort was 69.4%, which was the highest compared to that observed for other genes. Previous studies have shown that TP53 mutations in PSC are accompanied by other mutations, suggesting that mutated TP53 may not be the driver gene for PSC but rather augment genomic instability [12]. Since multiple gene mutations drive tumor growth, recovery of TP53 function can trigger apoptosis and clear the tumor cells [13].

In our study, the frequency of KRAS mutation was 30.6%, which is consistent with the reported mutation rate of 15%-30% in cancer. The PSC patients harboring KRAS mutations had worse overall survival compared to those with wild-type KRAS. Another study conducted on 46 cancer patients found that KRAS mutations increased the risk of metastasis, recurrence and death [14]. In addition, Fallet et al. [15] showed that all cancer patients with KRAS mutation have the wild-type EGFR, which was also observed in our study. However, the underlying mechanism needs to be further clarified. Nevertheless, the frequency of EGFR mutations in cancer patients is ambiguous. For instance, while one study reported that EGFR mutations occur in 9% of cancer patients [16], another study conducted on the Asian population found the mutation frequency of EGFR was 20% [17]. In our study, the frequency of EGFR mutations in the PSC patients was 19.4%, which was similar to the data on the Asian population. This suggests that the EGFR mutation may be closely related to race and ethnicity. Although tyrosine kinase inhibitors targeting EGFR have shown encouraging results, there is no data to support their efficacy against PSC.

Exon 14 skipping in MET occurs in about 3% of the NSCLC patients. Recent studies have shown that MET mutations are more common in sarcomatoid lung cancer than NSCLC [18, 19]. One study reported MET mutation frequency of 22.2% (8/36) among PSC patients [20], which was significantly higher than the 13.9% observed in our study. This can be attributed to differences in ethnicity and the small sample size. Other small sample studies and case reports have demonstrated that c-Met inhibitors (such as crizotinib and capotinib) may be effective against tumors with exon 14 jumping mutations of MET. Since none of patients in our cohort were treated with c-Met inhibitors, we cannot determine their efficacy in patients with PSC. We also detected mutations in PKD1, THADA, RB1, NF1, PTCH1, BRCA1 and BRAF, of which BRCA1 mutations were prognostically relevant, although little is known regarding their role in PSC.

In conclusion, the overall survival of the KRAS wild-type PSC patients was better compared to those harboring the KRAS mutations. Thus, detection of KRAS mutations can not only guide targeted therapy, but also predict prognosis. Although BRCA1 gene mutation was also identified as a prognostic factor, given the small sample size and racial differences, the result may have been biased and thus needs to be clarified in future studies. Therefore, it is worth conducting NGS using blood or tumor tissue specimens to detect germ-line mutations, and provide a basis for diagnosis and individualized treatment.

## Data Availability

All data generated or analyzed during this study are included in this published article.
